# Human Bocavirus in Children

**DOI:** 10.3201/eid1205.051523

**Published:** 2006-05

**Authors:** Vincent Foulongne, Michel Rodière, Michel Segondy

**Affiliations:** * Montpellier University Hospital, Montpellier, France

**Keywords:** Bocavirus, children, respiratory tract disease, letter

**To the Editor:** Respiratory tract infection is a major cause of illness in children. Despite the availability of sensitive diagnostic methods, detecting infectious agents is difficult in a substantial proportion of respiratory samples from children with respiratory tract disease (*1*). This fact suggests the existence of currently unknown respiratory pathogens.

A new virus has been recently identified in respiratory samples from children with lower respiratory tract disease in Sweden (*2*). Analysis of the full-length genome sequence showed that this virus is closely related to bovine parvovirus and canine minute virus and is a member of the genus *Bocavirus*, subfamily *Parvovirinae*, family *Parvoviridae*. This virus has been provisionally named human Bocavirus (HBoV) (*2*). HBoV in respiratory samples from Australian children was also recently reported (*3*). Involvement of this new virus in respiratory tract diseases merits further investigation. We have therefore retrospectively tested for HBoV nasopharyngeal samples collected from children <5 years of age hospitalized with respiratory tract disease.

Samples were collected from 262 children from November 1, 2003, to January 31, 2004. The samples were tested for respiratory viruses by using direct immunofluorescence assays with monoclonal antibodies to respiratory syncytial virus; influenza virus types A and B; parainfluenza virus types 1, 2, and 3; and adenovirus. Samples were also placed on MRC5 cell monolayers for virus isolation and tested for human metapneumovirus by reverse transcription–polymerase chain reaction (RT-PCR). Nucleic acids were extracted from samples, stored at –80°C, and tested for HBoV DNA by PCR with primers specific for the predicted NP1 gene as previously described (*2*). The expected product size was 354 bp. In each experiment, a negative control was included, and positive samples were confirmed by analyzing a second sample. Amplification specificity was verified by sequencing.

Nine (3.4%) samples were positive. Comparison of PCR product sequences of these 9 isolates (GenBank accession nos. AM109958–AM109966) showed minor differences that occurred at 1 to 4 nucleotide positions, and a high level of sequence identity (99%–100%) was observed with the NP1 sequences of the previously identified ST1 and ST2 isolates (*2*). This finding indicates that HBoV is a highly conserved virus.

HBoV was the only virus identified in 6 children and was associated with respiratory syncytial virus in 3 other children. An infection with other respiratory viruses was detected among 153 (60.5%) of the 253 HBoV-negative children. The viruses identified were respiratory syncytial virus in 114 (43.5%) samples, human metapneumovirus in 27 (10.3%) samples, influenza A virus in 14 (5.4%) samples, rhinovirus in 4 (1.5%) samples, adenovirus in 2 (0.8%) samples, and parainfluenza virus type 3 in 1 (0.4%) sample. Respiratory syncytial virus was associated with human metapneumovirus in 9 (3.4%) samples.

Clinical characteristics of the HBoV-infected children are shown in the Table. Children infected with only HBoV had mild-to-moderate fevers. Leukocyte counts and C-reactive protein levels were normal or moderately elevated. Chest radiographs obtained for 7 children showed abnormalities such as hyperinflation and interstitial infiltrates. Bronchiolitis was the major diagnosis. Dyspnea, respiratory distress, and cough were the most common respiratory symptoms observed. Four (44%) HBoV-infected children were born preterm, which suggests that these children have an increased susceptibility to HBoV-associated diseases. All children recovered and were discharged within 1 to 6 days.

**Table:** Clinical characteristics of children infected with human Bocavirus*

Age (mo)	Sex	Copathogen	Fever (°C)	Leukocytes (× 10^3^/μL)	CRP (mg/L)	SaO_2_ (%)	Underlying condition (wks of pregnancy)	Diagnosis	Symptoms†
8	M	RSV	39.0	NA	NA	NA	None	Bronchiolitis	D, C
39	M	RSV	38.5	14.6	13.0	95	Preterm (36)	Asthma	RD, D
12	F	RSV	37.5	15.9	13.6	NA	None	Bronchiolitis	RD, C, O
19	F	None	37.3	15.6	<5.0	91	Preterm (35)	Bronchiolitis	RD
8	M	None	36.8	NA	NA	95	None	Bronchiolitis	D
10	M	None	38.2	12.6	9.6	NA	Preterm (28)	Bronchiolitis	D
9	F	None	38.5	12.7	<5.0	68	Chronic respiratory disease	Acute respiratory distress	RD
14	M	None	38.1	9.0	38.5	93	None	Bronchiolitis	RD, D, C
11	M	None	37.8	9.4	<5.0	96	Preterm (31)	Asthma	D

*CRP, C-reactive protein; SaO_2_ saturation of arterial oxygen; RSV, respiratory syncytial virus; NA, not available. †D, dyspnea; C, cough; RD, respiratory distress; O, otitis.

The 3.4% incidence of HBoV observed in our study is similar to that (3.1%) reported by Allander et al. (*2*). HBoV was the only infectious agent identified in 6 children, which suggests that it was the causative agent of the disease. However, more studies conducted in children with and without respiratory disease as well as in adults and elderly persons are needed to better assess the pathogenic role of HBoV.
